# Desensitized gamers? Violent video game exposure and empathy for pain in adolescents – an ERP study

**DOI:** 10.1080/17470919.2023.2284999

**Published:** 2023-12-06

**Authors:** Ewa Miedzobrodzka, Johanna C. van Hooff, Lydia Krabbendam, Elly A. Konijn

**Affiliations:** a Department of Communication Science, Media Psychology Program, Faculty of Social Sciences, Vrije Universiteit Amsterdam, Amsterdam, the Netherlands; b Department of Clinical, Neuro-, and Developmental Psychology and Institute of Brain and Behavior Amsterdam, Faculty of Behavioral and Movement Sciences, Vrije Universiteit Amsterdam, Amsterdam, the Netherlands; c College of Life Sciences, Faculty of Science, University of Amsterdam, Amsterdam, the Netherlands

**Keywords:** Violent video games, adolescents, empathy for pain, ERP, desensitization

## Abstract

This Event-Related Potential (ERP) study aimed to test how habitual and short-term violent video game exposure (VVGE) may affect empathy for pain responses in adolescents. In a within-subjects design, boys (*N* = 56; aged 12–16 years) performed a pain judgment task before and immediately after playing a violent video game. In this task, participants judged whether photos of hands depicted on their screen were in a painful situation or not. While both the P3 and the LPP components were not related to habitual violent video game exposure, general exposure to antisocial media content predicted lower P3 amplitudes to painful pictures. Further, 40 min of violent gameplay did not affect the P3 responses; however, it temporarily decreased LPP responses to painful pictures, suggesting a modest short-term desensitization effect. However, this latter interpretation is limited by a strong LPP pain effect – a significant amplitude difference between painful and non-painful pictures – that remained present in the post-game condition. Such persistent LPP effect may relate to the notion that adolescents are still learning how to properly regulate their emotional reactions. This study contributes to the limited literature on violent video games’ desensitization in adolescents’ brains, opening new avenues for media violence research.

Violent video games often depict the suffering of virtual characters; thus, frequent exposure to such games may affect empathy for pain in players. A recent Event-Related Potential (ERP) study indicated that only 40 min of violent gaming is enough to reduce empathy for pain reactions in young adults’ brains who had no prior experience with playing such games (Miedzobrodzka et al., 2022). The study further showed that habitual violent gaming exposure was related to lower empathy for pain responses. It is yet unclear, however, whether similar results might also be found in adolescent boys who are often avid game players (Rideout, 2015) and whose empathy for pain skills are still developing (Mella et al., 2012). Therefore, by applying a similar within-subjects design as in Miedzobrodzka et al. (2022), we investigated whether habitual as well as short-term exposure to violent video games may decrease empathetic brain responses in adolescent players, indicating desensitization.

It is important to focus on such young gamers, since they could be especially susceptible to violent media effects (Crone & Konijn, 2018; Konijn & Achterberg, 2020). Adolescence is considered a sensitive period for social, cognitive, and emotional development (Crone & Dahl, 2012), including empathy for pain (Mella et al., 2012). It is also a period in which teenagers experience problems with controlling emotions since their emotion regulation skills are still developing (Ahmed et al., 2015). Moreover, teenagers have been found to be especially susceptible to the impact of negative emotional stimuli (Cohen-Gilbert & Thomas, 2013), to be hyper-sensitive to rewards (Telzer, 2016), and to be more prone to risk-taking behavior (Blakemore & Robbins, 2012). All these factors may attract adolescents to playing violent video games and make them susceptible to violent video game effects (Konijn & Achterberg, 2020).

This susceptibility was confirmed in a meta-analysis showing that adolescents who regularly played violent video games were at a greater risk of developing aggressive behavior over time (Prescott et al., 2018). Another recent meta-analysis indicated that especially adolescents aged around 14 years of age could be at risk from the negative effects of violent video games on aggression (Burkhardt & Lenhard, 2022). Such risk is related to the increased frequency of (violent) video gaming around this age (Greenberg et al., 2010), and to the peak of aggressive behavior in teenagers, explained by hormonal changes in the adolescent body (Carré et al., 2017). Given these meta-analytic findings, concerns of public opinion, especially parents, about the potential negative effects of violent video games on young players seem justified.

## Violent video game desensitization

Exposure to violent video games may affect different social skills, cognitive and emotional processes which may underlie aggression (Ferguson & Konijn, 2015). An important effect frequently studied in this context is desensitization. According to the Media Violence Desensitization Model (Carnagey et al., 2007), repeated exposure to violent video games may result in desensitization observed as decreased affective reactions to initially aversive stimuli, such as painful and distressing pictures, which in turn could increase antisocial and decrease prosocial behavior.

Violent video game desensitization has been studied in the brain using various tasks, most frequently involving passive viewing of different visual stimuli, such as realistic violent IAPS pictures (Engelhardt et al., 2011) or cartoon-like drawings of painful situations (Szycik et al., 2017). Further, studies on desensitization involved different study designs: cross-sectional, which examined associations with habitual exposure to violent games (Bartholow et al., 2006) or experimental, which tested short-term results of exposure in an experiment (Engelhardt et al., 2011). Finally, they applied different neuroimaging techniques to observe desensitization. In fMRI research, desensitization as a result of exposure to violent media content has been observed in several studies as reduced neural activity in emotional brain areas such as the amygdala, anterior insula, and anterior cingulate cortex (Guo et al., 2013; Montag et al., 2012; Weber et al., 2006). However, particularly more recent fMRI studies failed to obtain evidence for such desensitization effect (Gao et al., 2017; Kühn et al., 2018; Szycik et al., 2017). In contrast, the majority of ERP studies *in adults* have found a desensitization effect as a result of - (Engelhardt et al., 2011; Miedzobrodzka et al., 2022) or related to – violent video game exposure (Bartholow et al., 2006; Bailey et al., 2011; Stockdale et al., 2017; Miedzobrodzka et al., 2022; but see; Goodson et al., 2021 for non-significant findings). In most of these studies, such desensitization effect was observed as a reduced P3 amplitude in response to emotionally negative pictures suggesting that less attentional resources were directed toward the processing and evaluation of these pictures.

In the context of the current research, the most relevant of these studies is the work by Miedzobrodzka et al. (2022), which used pictures of hands in painful situations (e.g., cutting one’s finger) to measure empathy for pain responses (Coll, 2018). In that study (Miedzobrodzka et al., 2022), ERP empathy for pain reactions of adult males with different levels of habitual exposure to violent video games were measured before as well as immediately after playing a violent video game. This design enabled the observation of an interaction between habitual and short-term exposure to violent video games and related to that – different types of desensitization. *Before* playing the game in the lab, participants with no habitual violent video game exposure (VVGE) had higher P3 amplitudes for the painful pictures than for the non-painful ones, reflecting a pain effect. A similar pain effect was not present at that time point in participants with high VVGE levels (above 8 hours of violent gameplay/week), suggesting *habitual desensitization* in frequent players. Furthermore, when comparing brain reactions before versus after gameplay, a significant decrease in P3 amplitudes for painful pictures was found, but only in participants with no habitual VVGE, suggesting *short-term desensitization* in this group. Such an interaction between habitual VVGE and experimental exposure was in line with earlier ERP findings (Engelhardt et al., 2011), and fMRI investigation (Gentile et al., 2016).

If violent video games would affect adolescents in a similar way as in the aforementioned adult ERP studies, it could have pronounced consequences for the future life of young gamers, because it may predict antisocial behavior (Engelhardt et al., 2011) and limit the development of proper social skills. Therefore, it is important to examine to what extent exposure to violent video games may be related to desensitization in adolescent gamers. Until now, the literature on violent video game desensitization in teenagers is very limited (Brockmyer, 2022) and most of the earlier research in this context, as described above, was focused on adult samples.

Therefore, in the current work, we aimed to fill this research gap by studying possible desensitization of adolescents’ empathy for pain responses in the brain, which can be affected by exposure to violent video games. We applied an ERP approach, which can provide time-sensitive information about implicit underlying processes of a desensitization effect. In order to introduce our study, we first review literature related to the measurement of brain reactions to empathy for pain with ERPs. Then, to explain how adolescents may react to painful pictures, we briefly review the literature on empathy for pain development.

## Empathy for pain in the brain

“Empathy” is a broad term and a complex psychological construct, as well as a skill crucial for social interactions (Fan et al., 2013). While accurate empathic reactions are a basis of good social functioning, problems with empathizing may underlie antisocial behaviors, as illustrated in people characterized with psychopathy (Baron-Cohen, 2012) or diagnosed with Conduct Disorder (Decety et al., 2009; Michalska et al., 2015). In the context of the current study, it is particularly relevant to focus specifically on *empathy for pain* which refers to reactions when observing the suffering of another person (Coll et al., 2017). Such reactions could include feelings of concern, distress, sympathy, compassion, and pro-social behavioral tendencies (Goubert et al., 2005), which can also be observed in the brain.

In fMRI studies, seeing others in pain evokes activation of brain areas (medial/anterior cingulate cortex and bilateral anterior insula) which are also triggered when experiencing pain oneself (Lamm et al., 2011). Further, observing the suffering of others in a painful situation elicits higher ERP amplitudes as compared to viewing others in a non-painful situation, reflecting a “pain effect” (Coll, 2018; Fan & Han, 2008). This ERP pain effect typically consists of several components that are separated in time (Coll, 2018), confirming the theoretical dual-process model of empathy for pain (Goubert et al., 2005). According to this model (Coll, 2018; Fan & Han, 2008), observing suffering of others evokes an “early” empathy for pain reaction which involves automatic, stimulus-driven affective responses, such as emotional contagion, which are reflected in early ERP components, such as the N2. Furthermore, “late” empathy for pain responses could be observed in the subsequent P3 and Late Positive Potential (LPP), which are associated with more cognitive-evaluative reactions to empathy for pain (Coll, 2018). The P3 component is typically observed 300–500 ms after stimulus onset and the LPP component between 400–800 ms post-stimulus. While these two components partially overlap (Hajcak & Foti, 2020) and represent the same class of late ERP components (MacNamara et al., 2022), they are associated with two different processes in response to viewing painful pictures: aversive arousal (P3) and emotion regulation

(LPP).

In the ERP literature, P3 amplitudes are found to be sensitive to salient stimuli evoking aversive emotional reactions (Hajcak et al., 2012; Polich, 2007). More specifically, in the context of empathy for pain, *higher* P3 amplitudes may suggest *higher* emotional arousal related to viewing painful stimuli (Coll, 2018). In the ERP literature, the LPP amplitudes have been linked to top-down emotion regulation or emotion suppression processes (Decety et al., 2010; Dennis & Hajcak, 2009; Ikezawa et al., 2014; MacNamara et al., 2022; Schipper & Petermann, 2013), also in adolescent samples (Desatnik et al., 2017). In the context of empathy for pain (Coll, 2018), *higher* LPP amplitudes may indicate *less* effective regulation/suppression of initial emotional arousal (Mella et al., 2012).

In the current research, we focused on these two late ERP components given that both P3 and LPP components can be indicators of empathy for pain responses (Coll, 2018; Fan & Han, 2008; Ikezawa et al., 2014), and which were found to be affected by exposure to violent video games (Bartholow et al., 2006; Engelhardt et al., 2011; Miedzobrodzka et al., 2022). Depending on the task context, lower P3 and lower LPP amplitudes may indicate different aspects of desensitization related to exposure to violent video games. That is, lower P3 amplitudes might reflect a decrease in emotional reactivity and lower emotional arousal in response to viewing violent or painful pictures (Engelhardt et al., 2011). Decreased P3 amplitudes have been observed in relation to habitual VVGE (Bartholow et al., 2006; Miedzobrodzka et al., 2022) and as a result of short-term exposure to a violent video game as well as an outcome of an interaction between habitual and short-term exposure to violent video games (Engelhardt et al., 2011; Miedzobrodzka et al., 2022). Further, initial ERP findings suggested that a comparable desensitization effect was also present for a late P625 (LLP-like) component in adults (Miedzobrodzka et al., 2022), possibly associated with more effective emotional suppression.

However, until now, no study has tested whether such desensitization effects may also be observed in younger players, whose empathy for pain and emotion regulation skills are still developing (Mella et al., 2012).

## Empathy for pain in the adolescent brain

The ability to empathize with others in pain is developing from early childhood (Cheng et al., 2014) through adolescence until adulthood (Greimel et al., 2010; Mella et al., 2012). Empathy development involves a transition from automatic emotional sharing to more controlled cognitive responses, involving emotion regulation and assessment of the situation (Decety & Jackson, 2004; Preston & de Waal, 2002). Neuroimaging studies indicated that during infancy very early empathetic responses (such as emotional contagion) are based on rudimentary and sensory networks (Levy et al., 2018). In contrast, empathetic responses in late adolescence and young adulthood are more controlled than in infancy because they involve frontal brain areas (Decety et al., 2011). The developmental changes in the empathy for pain reactions are observed in parallel with the development of emotion regulation skills (Desatnik et al., 2017) which are indicated by the maturation of frontal brain areas and networks involved in emotion regulation (Pozzi et al., 2021). Exposure to violent video games may affect empathy for pain reactions and related emotion regulation processes in adolescents (Konijn & Achterberg, 2020). As far as we know, no ERP studies with adolescents were reported in this respect.

## The current study

Following Miedzobrodzka et al. (2022), in this study, we understand desensitization as *decreased empathy for pain in brain responses*: lower brain reactions (ERP amplitudes) when observing the suffering of another person. Based on the ERP meta-analytical findings (Coll, 2018) and previous ERP studies on violent video games’ desensitization in adult players (Bartholow et al., 2006; Engelhardt et al., 2011; Miedzobrodzka et al., 2022), we focused on P3 and LPP empathy for pain responses in adolescent gamers (aged 12–16 years). We aimed to investigate whether P3 and LPP empathy for pain ERP responses could be affected by both short-term and habitual exposure to violent video games in adolescent gamers. In general, we expected that exposure to violent video games would affect the P3 and LPP amplitudes for painful stimuli in a similar way, since they belong to the same class of late ERP components (MacNamara et al., 2022) and since they are both associated with the late cognitive-evaluative empathy for pain response (Coll, 2018; Fan & Han, 2008; Ikezawa et al., 2014). We focused on adolescent boys given that they are avid players of violent games (Rideout, 2015), and because adolescence is a sensitive period for social skills development (Blakemore & Robbins, 2012), including empathy for pain (Levy et al., 2018; Mella et al., 2012).

Following the within-subjects ERP approach of Miedzobrodzka et al. (2022), we tested our adolescent participants twice: once before and once immediately after playing a violent video game for 40 min, while considering their habitual exposure to violent video games as a possible moderator. Such a mixed quasi-experimental design allowed studying an actual within-person change in neural empathy for pain responses due to violent gameplay, and to take into account individual differences in empathy for pain reactions, as well as to consider between-participant differences in violent gaming habits. Based on this earlier ERP study, we preregistered (https://aspredicted.org/blind.php?x=wu33f5) the following hypotheses:

H1: Painful pictures would be rated as more painful and elicit higher ERP amplitudes (P3 and LPP) compared to non-painful pictures, reflecting an ERP pain effect.

H2: Habitual VVGE would be related to lower ERP amplitudes (P3 and LPP) to painful pictures (reduction of a pain effect), indicating habitual desensitization.

H3: Playing a violent video game in an experimental setting would result in lower ERP amplitudes (P3 and LPP) to painful pictures as compared to the pre-game measurement, indicating short-term desensitization.

H4: Habitual VVGE and short-term exposure to a violent game would modulate ERP amplitudes (P3, LPP) to painful pictures. Lower VVGE levels would be related to short-term desensitization after the game, and higher VVGE levels would be related to habitual desensitization before the game.

In addition, we explored for potentially moderating effects of two individual characteristics: age and general exposure to antisocial media content (beyond violent video games). We tested for participants’ age to assess to what extent developmental changes in empathy for pain (Mella et al., 2012) might contribute to expected effects and to explore whether younger players may be more susceptible to the effects of violent gaming than older players (Crone & Konijn, 2018; Konijn & Achterberg, 2020). Further, since adolescents may be exposed to antisocial media content in other media types than video games, antisocial media exposure might be a better predictor of violent media effects on adolescents than habitual VVGE (Miedzobrodzka et al., 2022). Therefore, we tested exposure to antisocial media content as an alternative moderator of the interaction between the picture type factor (Pain vs. no Pain) and pre- vs. post-game condition comparison (Time). Given the limited literature on violent video game desensitization in adolescents (Brockmyer, 2022) and developmental changes in empathy for pain (Mella et al., 2012), we treated those analyses as exploratory.

## Method

### Participants

This study involved male adolescents (*N* = 56) aged between 12 and 16 years (*M*_*ag*e_ = 13.74 years; *SD*_*ag*e_ = 1.13), 92.9% Dutch. They were recruited from two schools in urban areas in The Netherlands. The minimal estimated *N* to observe a pain effect (Coll, 2018) and a desensitization effect in ERP studies was 46 participants (see details in our preregistration: https://aspredicted.org/blind.php?x=wu33f5 and inclusion criteria in Supplementary Materials). Both participants and their parents provided active consent. Participants were rewarded with a gift card of 20 EUR after completing the procedure. The study was approved by the Institutional Ethical Review Board (approval #VCWE-2018-173).

### Design

The current study is a quasi-experiment, including two within-participants factors: Time (pre-game vs. post-game) and Pain (painful pictures vs. non-painful pictures). Participants’ habitual exposure to violent video games (VVGE) is tested as a continuous moderator of the pain effect, as well as for the Time x Pain interaction. With such a design, we were able to measure an actual change in brain responses to painful pictures before vs. after playing a violent game, while accounting for individual differences in habitual exposure to such games.

### General procedure

Adolescents and parents received an invitation letter to the study from their school. After filling in an online recruitment form and checking if they met the inclusion criteria, participants and their parents received more information about the EEG procedure and were contacted by a research assistant to schedule an appointment. Upon coming to the EEG lab, participants received further information about the study procedure and gave their consent to participate. First, participants completed an online survey (10 min) measuring their video game experience and antisocial media content exposure, three traits (empathy, physical aggressiveness, and sensation-seeking), and their demographics. Next, they were connected to the EEG equipment, performed the first pain judgment task (10 min), and played a violent video game (40 min). Subsequently, they answered six questions about their experience with the game and next performed the pain judgment task for the second time (10 min). After the task, they were disconnected from the EEG, debriefed, and rewarded. The whole procedure lasted about 2 hours.

### Individual characteristics

#### Habitual Violent Video Game Exposure (VVGE)

Participants named three favorite video games that they played most frequently and reported how often they played these video games (hours/week) in the past 6 months (Fikkers et al., 2016). Following Pan European Game Information (PEGI; https://pegi.info/), video game age and violent content ratings were used to classify video games as violent or nonviolent (Busching et al., 2015). Every game with an age label 12+, 16+, and 18+ and a violent content label was coded as a violent one. Every video game with an age label 3+, 7+, and 12+ and without a violent content label was coded as a nonviolent game (Miedzobrodzka et al., 2022). Violent Video Game Exposure (VVGE) was computed by summing the time of exposure (hours/week) spent on a violent video game. In a similar way, an indicator of non-Violent Video Game Exposure (non-VVGE) was computed.

#### Antisocial media exposure

Antisocial media exposure was measured with a subscale (12 items; α = .91) from the Content-based Media Exposure (C-ME) questionnaire by den Hamer et al. (2017), validated for adolescent samples. Item example: “How often do you watch, on the Internet/TV/games/mobile phone/DVD, people who shoot at another person”. Scale ranged from 1 (*never*) to 5 (*very often*).

See Supplementary Materials for details of the measurement of individual characteristics: traits empathy, aggressiveness and sensation seeking, video gaming habits, and video gameplay experience check, including levels of perceived game violence, frustration, excitement, engagement, interest, and challenge.

### Violent video gameplay

In between the two pain judgment tasks, participants played a first-person shooter (FPS) game *Call of Duty: Modern Warfare 3* for 40 min. Participants played it on a PlayStation 3 with a game controller and headphones. They were seated at about 80 cm from a TV screen (size 24’’). The difficulty level was set to regular. Inexperienced gamers received additional instruction regarding how to use the game controller. All participants were informed beforehand about the game content. This game is 18+ rated by PEGI as it contains “extreme violence, violence towards defenseless people and strong language”. The game is highly popular among gamers (Nielsen, 2017) and was used as a manipulation in previous research on violent video gaming effects (Grizzard et al., 2016; Miedzobrodzka et al., 2022).

### The pain judgement task

**Figure 1.**
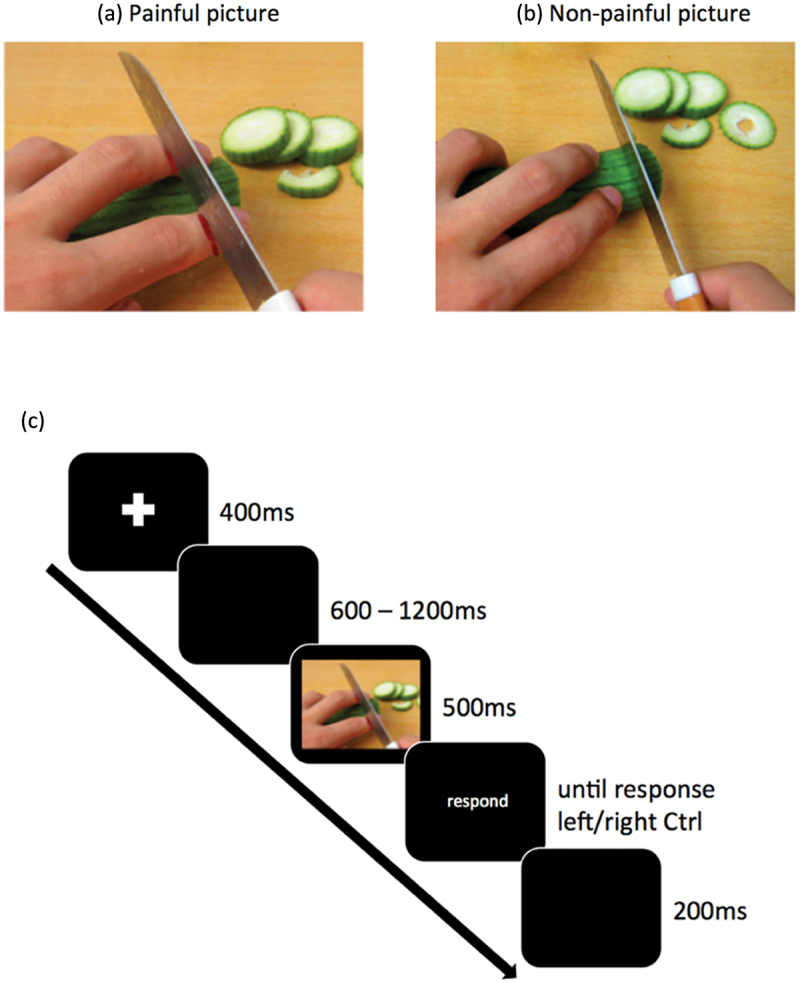
Example of stimuli used in the task (Meng et al., 2012; Meng et al., 2023) (A and B); example of an EEG trial in the pain judgment task (C).

*Note*. Each trial started with a fixation cross (400 ms), followed by a black screen interval of 600–1200 ms (in steps of 200 ms) in order to minimize time-locked anticipatory processes. In the ERP blocks, the pictures were presented for 500 ms in a randomized order (Ikezawa et al., 2014) and in the behavioral rating block – for 1000 ms. Picture presentation was followed by a black screen with a white “respond” word in the middle until a response was given. Before the start of the next trial, a black screen appeared (200 ms).

Participants viewed pictures of hands in painful or non-painful everyday situations (e.g., cutting a cucumber) while their EEG was recorded. The pain judgment task consisted of four blocks separated with a 30 s break. The first block was a short training block (6 trails) with a different set of pictures (Canizales et al., 2013) but with similar instructions as in the subsequent two blocks. In the second and the third block, participants viewed 96 pictures of hands (48 trials per block; Meng et al., 2012; Meng et al., 2023; Figure 1) and were asked to decide if a picture was painful (press right “Ctrl” button) or non-painful (press left “Ctrl” button), while their EEG responses were recorded. In the last and fourth block, measuring behavioral pain ratings but no EEG, the participants viewed the same 48 pictures, but this time they were asked to rate them on a 1–6 scale (1 = no pain; 6 = very intensive pain).

### EEG recording and pre-processing

EEG data were recorded from 64 sintered (AG/AgCl) electrodes, positioned at standard International 10/20 System locations using the Biosemi ActiveTwo system (Biosemi, Amsterdam, the Netherlands). Additionally, six external electrodes of the same type were used to record the electro-oculogram (EOG) and to serve as a reference. Two electrodes were placed 2 cm above and below the left eye to record the vertical EOG (VEOG) and two electrodes were placed 1 cm lateral to the external canthi to record the horizontal EOG (HEOG). Two electrodes were placed at the mastoids and an average from these two electrodes was used as an offline reference (after the EEG recording), which is common in EEG research using BioSemi equipment (Leuchs, 2019). Data were recorded with a sample rate of 256 Hz.

Data were processed with EEGLAB (Delorme & Makeig, 2004) and ERPLAB Toolboxes (Lopez-Calderon & Luck, 2014) in Matlab (2017). EEG data were filtered with a band-pass filter (0.05–30 Hz, roll-off 48/160 dB), and epoched 200 ms before and 1000 ms after stimulus onset, with a baseline correction of 200 ms before stimulus. A regression-based method of ocular correction was applied (Gratton et al., 1983). Epochs were removed with the automatic artifact rejection procedure if they were falling outside −120 μV to 120 μV range, or if they included voltage steps of more than 80 μV within 200 ms moving window in steps of 50 ms. Artifact rejection resulted in 11.4% rejected trials in the pre-game condition (11.3% painful pictures; 11.5% non-painful pictures) and 9.8% in the post-game condition (9.5% painful pictures; 10.1% non-painful pictures). Trials were averaged separately for the painful and non-painful picture types both before and after the gaming period, resulting in four ERP categories: (1) painful pre-game; (2) non-painful pre-game; (3) painful post-game; (4) non-painful post-game. Following our preregistration and ERP literature (Coll, 2018), ERPs of interest were based on averaged amplitudes of centro-parietal electrodes, where the pain effect for P3 and LPP can be best observed.

### Analysis plan

See details of our preliminary analyses including correlations with individual differences, behavioral pain ratings, and video gameplay experience checks in the Supplementary Materials. For our main analyses, we followed the preregistered analysis plan. Pain ratings and mean ERP amplitudes (P3 and LPP) were analyzed as dependent variables in repeated measures ANOVA with the following within-subjects factors: (1) Time (pre-game vs. post-game) and (2) Pain (painful vs. non-painful picture). Both VVGE and non-VVGE were added as continuous covariates in all main analyses. For exploratory analyses, we ran repeated measures ANOVAs with the same factors as in the main analysis and two additional covariates: age and general exposure to antisocial media content. First, we tested a model with VVGE and age as covariates, which was followed by a model including antisocial media exposure (and age in the second step) as a covariate. The detailed results of the exploratory analyses are reported in the Supplementary Materials. The final data set, together with the analysis code and SPSS output are available at: https://osf.io/eack9/.

## Results

### Individual characteristics

Most participants had prior experience with video games (see details in Supplementary Materials). On average, participants played violent video games for 9.46 hours/week (*SD* = 7.80; range 0–33 hours/week), and 1.88 hours/week in nonviolent games (*SD* = 4.59; range 0–30 hours/week). Details of the individual characteristics are presented in Table S1.

### Correlations

Correlations between individual characteristics and behavioral pain ratings are reported in the Supplementary Materials (Table S2). A correlation analysis indicated that neither VVGE nor non-VVGE was related to the gameplay experience check. Thus, participants experienced the game in similar ways, regardless of their habitual exposure to violent or nonviolent video games (see details in Table S3).

### Hypothesis testing

Due to software and/or EEG recording problems resulting in incomplete data sets or an insufficient number of artifact-free trials, analyses of the pain rating data were based on 52 participants, while the ERP analyses were based on data of 50 participants. See the details of participants’ exclusions in the Supplementary Materials.

#### Pain ratings

The repeated-measures ANOVA with Time and Pain as within factors, and with VVGE and non-VVGE as covariates, indicated a main effect of Pain: *F*(1,49) = 225.01, *p* < .001, η_p_^2^ = .82. The painful pictures were rated as more painful (*M* = 4.09; *SE* = 0.12) than the non-painful ones (*M* = 1.10; *SE* = 0.03), supporting H1 and validating the stimulus materials. However, we did not find a main effect of Time, neither any interaction effects of interest (see details in Table 1).

**Table 1.** Main and interaction effects for the behavioral pain rating.

Effect	*df*	*F*	*p*	η_p_^2^
1. Time	1, 49	0.10	.752	.002
2. Pain	1, 49	225.01	<.000	.821
3. Time x Pain	1, 49	0.12	.734	.002
4. Pain x VVGE	1, 49	0.31	.583	.006
5. Time x Pain x VVGE	1, 49	0.10	.757	.002

*Note*. VVGE = Violent Video Game Exposure.

#### ERP results

**Figure 2.**
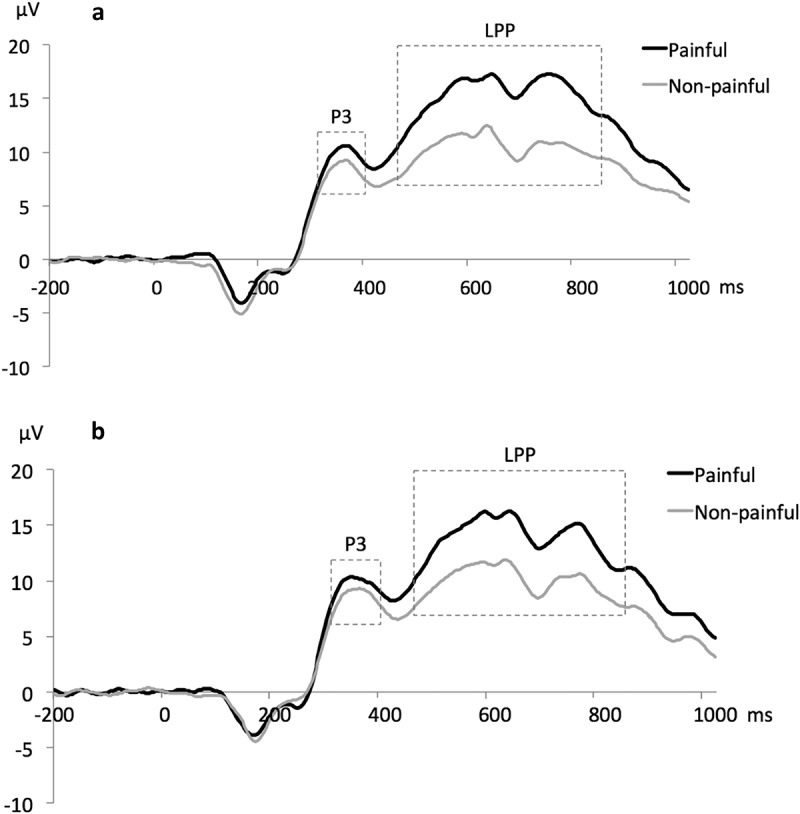
Grand average ERP waveforms averaged across 6 electrodes with marked time windows for the P3 and the LPP components: (A) pre-game condition, (B) post-game condition.

Based on the ERP literature (Coll, 2018; Meng et al., 2012), our preregistration protocol, and visual inspection of ERP waves and topographic maps, the following time windows for calculating mean amplitudes were set for centro-parietal electrodes: P3 at 300–400 ms and LPP at 450–850 ms. Averaged amplitudes across six centro-parietal electrodes (CP1, CPz, CP2, P1, Pz, P2; see Figure S1) were calculated for these time windows, which were used for subsequent analyses. Grand average ERP waveforms for these six electrodes (Figure 2) indicated a small pain effect for the P3 component, followed by a large pain effect for the LPP component over centro-parietal and parietal regions, which was in line with the ERP empathy for pain literature (Coll, 2018).

**P3**. A repeated-measures ANOVA with Time and Pain as within factors, and with VVGE and non-VVGE as covariates indicated no significant main effect of Pain: *F*(1, 47) = 3.54, *p* = .066, η_p_^2^ = .070, suggesting that the painful pictures elicited slightly but not significantly higher P3 amplitudes (*M* = 9.47; *SE* = 0.70) than the non-painful ones (*M* = 8.32; *SE* = 0.75). Further, we did not find a main effect of Time, nor a significant interaction effect of interest for the P3 component (Pain x VVGE – refuting H2, and Pain x Time x VVGE – refuting H4; see details in Table 2). Finally, we also found no Pain x Time interaction, related to H3.

**Table 2.** Main and interaction effects for the P3 component.

Effect	*df*	*F*	*p*	η_p_^2^
1. Time	1, 47	0.42	.522	.009
2. Pain	1, 47	3.54	.066	.070
3. Time x Pain	1, 47	0.46	.501	.010
4. Pain x VVGE	1, 47	0.42	.523	.009
5. Time x Pain x VVGE	1, 47	0.22	.644	.005

*Note*. VVGE = Violent Video Game Exposure.

**Figure 3.**
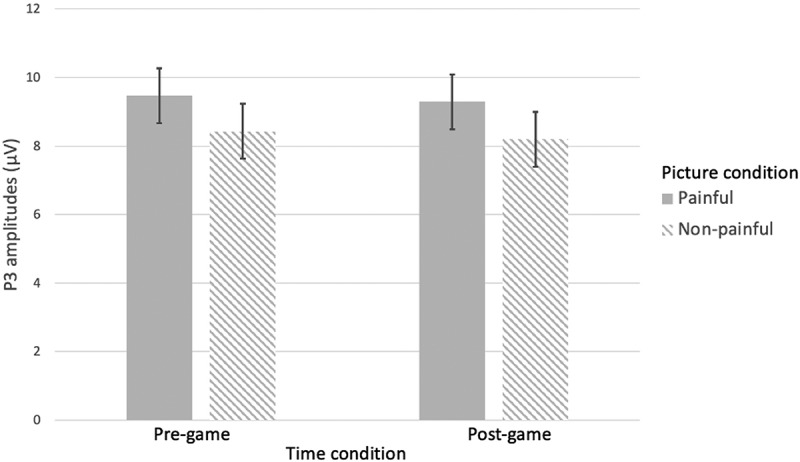
P3 amplitude as a function of Time (pre-game vs. post-game) and pain (painful vs. non-painful pictures).

*Note*. The main effect of pain was significant both in the pre-game and in the post-game conditions, but overall (across conditions) it was not significant (see text).

In order to understand why we did not find the Pain x Time interaction and to test H3 expecting a short-term desensitization effect, we further explored the pairwise comparisons with Bonferroni correction of the non-significant Pain x Time interaction (Figure 3). They indicated no change in amplitudes for painful pictures from pre- to post-game condition (*p* = .631), suggesting no short-term desensitization for the P3 amplitudes, rejecting H3. Moreover, no pre- to post-game changes were found for the nonpainful pictures (*p* = .729).

**LPP**. A repeated-measures ANOVA with Time and Pain as within factors, and with VVGE and non-VVGE as covariates, indicated a significant main effect of Pain: the painful pictures elicited higher LPP amplitudes (*M* = 14.43; *SE* = 0.77) than the non-painful ones (*M* = 10.04; *SE* = 0.74), *F*(1,47) = 70.20, *p* < .001, η_p_^2^ = .60. Moreover, a main effect of Time was also found: *F*(1,47) = 4.79, *p* = .034, η_p_^2^ = .093, reflecting a significant drop of LPP amplitudes (regardless of the picture type) from the pre-game (*M* = 9.04; *SE* = 0.80) to the post-gameplay measurement (*M* = 8.75; *SE* = 0.76). Finally, we did not observe any of the expected interactions (Pain x VVGE and Time x Pain x VVGE, rejecting H2 and H4 respectively). Finally, we also found no Pain x Time interaction, related to H3. See details in Table 3.

**Table 3.** Main and interaction effects for the LPP component.

Effect	*df*	*F*	*p*	η_p_^*2*^
1. Time	1, 47	4.79	.034	.093
2. Pain	1, 47	70.20	<.001	.600
3. Time x Pain	1, 47	0.02	.897	<.001
4. Pain x VVGE	1, 47	0.86	.358	.018
5. Time x Pain x VVGE	1, 47	0.09	.771	.002

*Note*. VVGE = Violent Video Game Exposure.

**Figure 4.**
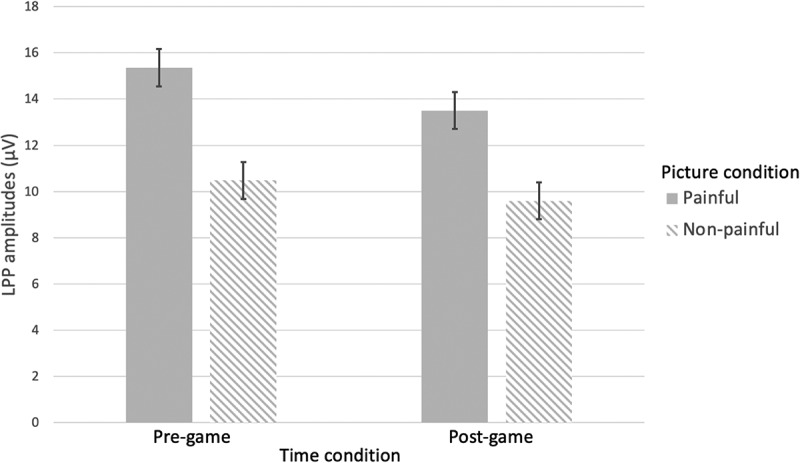
LPP amplitude as a function of Time (pre-game vs. post-game) and pain (painful vs. non-painful pictures).

*Note*. Significant effects of Pain and Time were found in both the pre- and post-gameplay measurements. In addition, a significant drop in LPP amplitude for painful pictures was observed from pre- to post-game measurements, while no significant changes were observed for the LPP amplitude for the non-painful pictures before and after playing the game.

To test H3, expecting a short-term desensitization effect, and to understand why we did not find the Pain x Time interaction, we further explored simple effects with the pairwise comparisons with Bonferroni correction of the non-significant Pain x Time interaction. They indicated a significant drop of the LPP amplitudes for painful pictures from the pre- to post-gameplay measurement (*p* = .031), suggesting a short-term desensitization and supporting H3. In contrast, the amplitudes for the non-painful pictures remained stable before vs. after the game (*p* = .220) (Figure 4).

### Exploratory analyses

In our preregistration, we also planned to run similar (mixed-model) ANOVA analyses for P3 and LPP with the high vs. low VVGE groups as the between-participants factor (instead of continuous VVGE and non-VVGE as covariates). However, given a skewed distribution of VVGE across the sample (Figure S1), which would result in a very unequal VVGE group division, we decided not to run follow-up analyses for the non-significant interactions with habitual VVGE as a continuous measure. Instead, we ran two exploratory ANOVA repeated measures analyses (with Pain and Time as within factors) with (1) VVGE and age as covariates and with (2) general exposure to antisocial media content (C-ME) as a covariate (and age added in the second step).

The results of the first exploratory ANOVA analysis with VVGE and age indicated no changes in the main results both for P3 and LPP; the effect of Pain, Time, and Pain x Time interaction were similar as in the main analyses. See details in Tables S5 and S6.

The results of the ANOVA analysis for P3 with C-ME as a covariate indicated no main effect of Time (*p* = .064), no Time x Pain interaction (*p* = .051), no Time x C-ME interaction (*p* = .071), and no Pain x C-ME interaction (*p* = 0.82; see details in Table 4). However, we found a significant 3-way interaction of Pain x Time x C-ME: *F*(1, 48) = 4.75; *p* = .034; η_p_^2^ = .090, suggesting that the level of habitual exposure to antisocial media content in general has moderated the Pain x Time effect. Adding age to the analysis as a second covariate, did not affect the Time x Pain x C-ME interaction (*p* = .024). See details in Table S7.

**Table 4.** Main and interaction effects for the P3 component with C-ME as a covariate.

Effect	*df*	*F*	*p*	η_p_^*2*^
1. Time	1, 48	3.61	.064	.070
2. Pain	1, 48	1.53	.222	.031
3. Time x Pain	1, 48	4.02	.051	.077
4. Time x C-ME	1, 48	3.42	.071	.067
5. Pain x C-ME	1, 48	0.06	.082	.001
6. Time x Pain x C-ME	1, 48	4.75	.034	.090

*Note*. C-ME = Content-based Media Exposure to antisocial media content.

**Figure 5.**
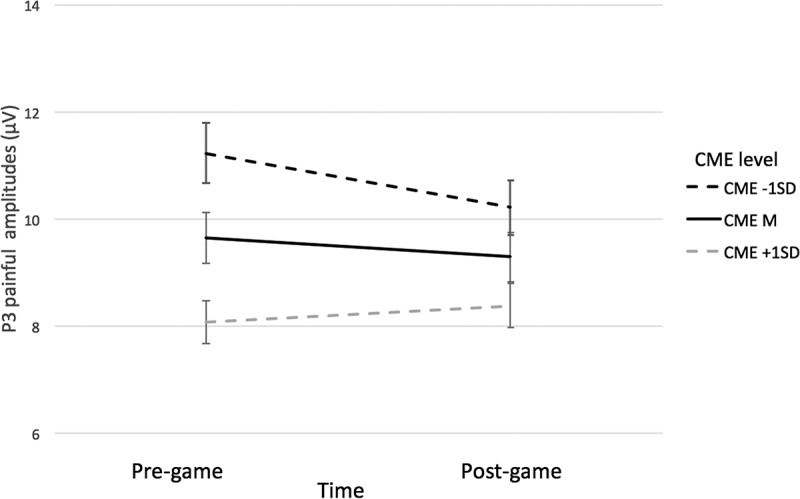
Visualization of the Time x pain x C-ME interaction for P3.

*Note*. C-ME moderated the level of P3 painful amplitudes before the game, but not after the game.

In order to further explore the Pain x Time x C-ME interaction, a MEMORE analysis (version 2.1, Montoya, 2019) was performed. In contrast to the above reported repeated measures ANOVA, MEMORE can test moderation for only one repeated-measures factor. Therefore, based on the Violent Media Desensitization Model (Carnagey et al., 2007) and violent media neural desensitization found in adults (Guo et al., 2013; Stockdale et al., 2017, 2015; Weber et al., 2006), we decided to focus on a possible moderation of C-ME on the P3 amplitudes for painful pictures before vs. after the game. In the MEMORE analysis (model 2; repeated measures moderation), we used the difference between P3 amplitudes for painful pictures pre-game vs. post-game (Y_1_ – Y_2_) as the dependent variable and C-ME as a moderator (W). For this analysis equation, see Supplementary Materials (p. 3). Results indicated that C-ME did not moderate the drop of the painful amplitudes before the game vs. after the game: *b* = −.91; *t*(48) = −0.90; *p* = .374; 95% CI [−2.96, 1.13]. However, in order to examine a possible C-ME desensitization, we checked the conditional effects which indicated that a higher level of C-ME predicted lower P3 amplitudes to painful pictures before the game: *b* = −2.20; *t*(48) = −2.06; *p* = .045; 95% CI [−2.34, −0.05]. It suggested a habitual desensitization related to higher exposure to antisocial media content. However, no such effect was found after the game: *b* = −1.28; *t*(48) = −1.17; *p* = .250; 95% CI [−3.50, 0.93]. See details in Figure 5.

The results of the analysis for LPP with C-ME as a covariate indicated a main effect of Pain (*F*(1,48) = 14.47; *p* < .001; η_p_^2^ = .232), and a trend of the main effect of Time: *F*(1,48) = 3.19; *p* = .080; η_p_^2^ = .062. No interaction with C-ME was found (see details in Table S8). Adding age as a second covariate to the ANOVA analysis for LPP resulted in a trend of Time x Pain x C-ME interaction (*p* = .094), however, no other main or interaction effects were found (see details in Table S9).

## Discussion

The aim of the current study was to investigate whether exposure to violent video games may affect empathy for pain reactions measured with ERPs in adolescent gamers. Specifically, we focused on the late “controlled” empathy for pain ERP responses: P3 and LPP and tested how they were affected by: (1) habitual VVGE, (2) short-term exposure to a violent gameplay, and (3) an interaction between habitual and short-term exposure to violent video games.

### Empathy for pain effect

First of all, the current behavioral and ERP findings indicated that the painful pictures were rated as more painful and elicited higher P3 and LPP amplitudes than the non-painful pictures, reflecting a pain effect and providing support for H1. This also stands in line with the notion that the pain judgment task is a valid method in evoking empathy for pain through brain reactions in late ERP components, such as the P3 or LPP (Coll, 2018). In line with the empathy for pain ERP literature (Coll, 2018; Fan & Han, 2008; Miedzobrodzka et al., 2022), we found that the P3 pain effect was less prominent than the LPP pain effect. Moreover, the observation of high LPP amplitudes for painful pictures is in line with empathy for pain results in adolescents, who had much higher LPP responses than adults. The relatively high LPP amplitudes in adolescents would reflect that they are not yet able to adequately downplay their emotions when viewing painful situations (Mella et al., 2012).

### Habitual violent video game exposure and desensitization

Our ERP findings indicated no Pain x VVGE interaction, which did not support H2, expecting a desensitization effect from habitual VVGE. In the main ANOVA analyses for the P3 and for the LPP, we did not find lower ERP empathy for pain responses associated with a higher VVGE level. Thus, our results for adolescents are not in line with previous ERP studies showing such habitual VVGE desensitization effect in *adult* gamers (Bartholow et al., 2006; Engelhardt et al., 2011; Miedzobrodzka et al., 2022; Stockdale et al., 2017). As an exception, our results are in line with Goodson et al. (2021) who conducted an ERP study in an adult sample in a similar design to ours. Goodson et al. (2021) categorized participants either as experienced players of violent video games or as those who had no such experience and found no P3 amplitude differences between these two groups for violent IAPS pictures. They explained the absence of a habitual desensitization effect by the suggestion that their participants might have experienced a general habituation to violent content in available media, besides video games (Grizzard et al., 2015). However, they did not measure general exposure to media-based violent content as we did (i.e., through the C-ME, den Hamer et al., 2017). Such an explanation might also be plausible for our study since our exploratory analyses indicated that exposure to antisocial media content in general could predict lower P3 responses to painful pictures. This may mean that not (only) habitual exposure to violent video games but exposure to antisocial content in all sorts of media (such as TV series and movies, digital games, online videos, social media) could moderate empathy for pain reactions of adolescents. Such a finding is in line with earlier research on recognizing emotional faces among adolescent players of violent video games (Miedzobrodzka et al., 2022), and the empathy for pain literature (Guo et al., 2013). However, since in the MEMORE analysis we did not find the Pain x Time x C-ME interaction as in the repeated measures ANOVA (only a conditional effect for Pain x C-ME in the before game condition). Therefore, this result should be interpreted with caution and requires further investigation.

Moreover, our ERP results for H2 (no Pain x VVGE interaction) appear to be different from earlier findings suggesting a habitual VVGE desensitization in adults (Bartholow et al., 2006; Engelhardt et al., 2011; Miedzobrodzka et al., 2022; Stockdale et al., 2017). This might be due to different developmental stages of adolescents and adults. As discussed before, empathy for pain skills (Mella et al., 2012) and emotion regulation skills (Desatnik et al., 2017) are still developing among adolescents, which has been observed in relatively high LPP amplitudes. Theoretically, such developmental changes may interact with possible habitual VVGE effects. On the one hand, the effects could be enhanced, given that adolescence is a sensitive developmental stage for media violence effects (Crone & Konijn, 2018), and emotion regulation skills are not yet fully matured (Desatnik et al., 2017). On the other hand, such effects might be decreased because empathy for pain is not yet fully developed (Mella et al., 2012). The best way to test these assumptions in future studies is through a longitudinal approach in adolescent samples.

Finally, possible habitual VVGE effects may depend on several factors, such as differences in years of (violent) gaming experience between adolescents and adults, and distribution of exposure to violent video games across a sample. On average, the tested adolescents played violent video games frequently (9.45 hours/week); however, it is nevertheless likely that they still have a shorter history of (violent) gaming in years than adults in comparable studies (cf. Miedzobrodzka et al., 2022). Although we could not make such a direct comparison between adolescents and adults, it is plausible that not only weekly gaming frequency but also gaming experience in years may account for possible differences in empathy for pain reactions of participants in the available studies. Such direct comparisons would be important to test in future studies.

Further, upon inspection of the habitual VVGE frequency distribution, we noticed that only six participants had never played violent video games before. Despite treating the habitual VVGE as a continuous measure and interest in general variance of habitual VVGE, recruiting more participants who are not experienced with violent video games might contribute to a greater variance in our data and would allow for a better between-participants comparison. For instance, another ERP study had more gamers without violent gaming experience (*n* = 15), yet with adults, which allowed to divide participants in VVGE-groups (no VVGE vs. high VVGE) and compare those groups in follow-up analyses (Miedzobrodzka et al., 2022). In our preregistration, we also aimed to run such follow-up analyses with an adolescent sample. However, in the present study, a skewed distribution of VVGE across the sample (Figure S1) would result in a very unequal VVGE group division. This did not allow for such extra analysis of the nonsignificant Pain x Time x habitual VVGE interaction.

### Short-term violent game exposure and desensitization

We did not observe a Pain x Time interaction for P3 nor for the LPP after the 40 min of violent video gameplay of our adolescent participants. This is in line with a recent quasi-experiment in a similar design by Goodson et al. (2021), who did not find changes in P3 responses to violent pictures in adults who played another *Call of Duty* series violent video game, but only for 10 min. However, we did find a small but significant drop in LPP amplitudes for painful pictures from pre- to post-gameplay measurement, suggesting a possible short-term desensitization effect, providing initial support for H3. This observation is in line with a previous study in young adults (Miedzobrodzka et al., 2022). Our findings for the LPP component indicated that 40 min of violent gameplay may thus be sufficient to affect adolescents’ top-down mechanisms that are involved in late “controlled” ERP empathy for pain responses related to enhanced emotion regulation (Dennis & Hajcak, 2009). This may mean that after playing a violent game, young players may have learned to (at least temporarily) suppress their empathetic responses, allowing them to be more efficient and focused on the purpose of the game. However, the extent to which we can be certain that the current data represent a clear short-term desensitization effect and support H3 is limited due to a large LPP pain effect, which remained significant both pre-game and post-game. This might be a reason for the non-significant Pain x Time interaction for the LPP component.

### Interaction between habitual and short-term exposure to violent video games

Finally, we have not observed a statistically significant interaction between Pain, Time, and habitual VVGE, which did not support H4, and which is not in line with previous research in adults (Engelhardt et al., 2011; Miedzobrodzka et al., 2022). These contrasting findings could be explained by the factors contributing to the lack of effect of habitual VVGE on empathy for pain reactions (H2): distribution of habitual VVGE and too few participants with no exposure to violent games. However, our results of the exploratory analyses suggested that exposure to antisocial media content may play a role in explaining the Pain by Time interaction. This latter result is in line with previous research by Grizzard et al. (2015) showing a general habituation to violent media content. Moreover, it suggests that exposure to antisocial content in various media, beyond violent video games, may better explain why participants who frequently watch antisocial media content may have showed lower responses to the painful pictures before playing the game. While this finding was just explorative and was found in the conditional analysis, it provides a promising line of thought for future research.

### Limitations and future directions

Our study has several strengths and limitations. A notable strength is applying a within-participants design allowing to account for individual differences in empathy for pain and to investigate an actual *change* of empathy for pain through brain reactions before vs. after playing a violent game. Moreover, our preregistered ERP study contributed to open science practices and transparency in violent video games research. However, several limitations should also be addressed. First of all, the lack of effects of habitual VVGE desensitization, in contrast to previous research (Miedzobrodzka et al., 2022) might be related to different distributions of habitual VVGE and too few participants without exposure to violent games in the current adolescent sample. Therefore, future studies could recruit participants that clearly differ in high vs. low habitual VVGE levels, or better, compare high vs. no habitual VVGE levels. This would preferably be done based on preregistered criteria and allow for a comparison of habitual VVGE differences between participants (Engelhardt et al., 2011).

Habitual exposure to violent video games may accumulate over time and overlap with empathy for pain development. Early adolescents, whose empathy for pain reactions are still developing, may have a too short history (in years) with habitual violent gaming to find its effects. In contrast, late adolescents or early adults might be exposed long enough to capture its effect when measuring brain activity. Hence, future longitudinal studies (either with an fMRI or ERP approach) could test whether habitual exposure to violent video games may have an impact on empathy for pain through brain responses over time, while controlling for individual developmental trajectories. Since adolescence is a sensitive period for social-cognitive development (Blakemore & Robbins, 2012), possible effects of violent video games on adolescents’ empathy for pain responses may have more serious consequences for future social skills in the life of teenage gamers.

The findings for the general exposure to antisocial content in all sorts of media (moderation of the Pain x Time interaction for P3) deserves further exploration in future studies. It could be related to both theoretical and methodological advantages of general media violence research over specific violent video game studies. Different studies used various measures of habitual VVGE, which makes direct comparisons very difficult and hampers the search for a most optimal way to measure habitual VVGE. In contrast, the C-ME (or C-ME2) is a validated measure of exposure to antisocial (and prosocial) media content (den Hamer et al., 2017). Therefore, future studies on the effects of violent media may also include this instrument.

Moreover, future experiments on violent video game effects should take into account that experience of video gameplay may vary across participants who may make different in-game choices and who may vary in terms of experience with playing video games. For instance, experienced gamers may make more progress in a game, while inexperienced gamers may make little progress or get stuck in a game. Such different experiences of gameplay may have different impacts on study outcomes. In the current research, we accounted for that by asking participants how they experienced the game in terms of the level of frustration, interest, engagement, game difficulty, and challenge. However, this could also be acknowledged by the study design, for example, by setting specific recruitment criteria such as only inviting participants who have a minimum of x-year experience with a given game.

A final limitation of the current study is that the full procedure took about 2 hours, including the gaming (40 min). Despite the relatively short duration of the EEG pain judgment task (two times 10 min), such a long experimental procedure could have contributed to the boredom or tiredness of our adolescent participants, which might have impacted their ERP responses after the game. This was indicated by the main effect of Time: after the game, LPP amplitudes of both painful and non-painful pictures were significantly dropped as compared to the pre-game measurement. A possible way to improve the procedure in the context of a within-participants design is to schedule two shorter lab sessions instead of one longer one or to schedule the survey at another time.

### Conclusion

In all, since our ERP study was the first to investigate the effects of violent video game exposure on empathy for pain in an adolescent sample, it provided new insights regarding developmental and neural mechanisms underlying social outcomes in young gamers. Our results only partially support the Violent Media Desensitization Model since we did not find habitual or short-term desensitization effects for the P3 component related to violent video gameplay, whereas habitual exposure to antisocial media content in general predicted lower P3 amplitudes to painful pictures. Furthermore, we observed temporarily decreased LPP responses to painful pictures after violent gameplay in the experiment, which may reflect a possible short-term desensitization effect. While such an adaptation of one’s responses may be helpful in a violent game environment, the extent to which it may affect social skills of adolescents, whose empathy for pain and emotion regulation skills are still developing, should be further studied in a longitudinal design.

## Supplementary Material

SNS-RP 32.23_Suppl_Mats_Clean.docx
