# Supplementary material for: Desensitized gamers? Violent video game exposure and empathy for pain in adolescents – an ERP study
Source: Soc Neurosci. 2023 Dec 6;18(6):365–81. doi: 10.1080/17470919.2023.2284999 (PMC10721224; doi:10.1080/17470919.2023.2284999)
Supplement: SNS-RP 32.23_Suppl_Mats_Clean.docx [file PSNS_A_2284999_SM0136.docx]

**Supplementary Materials**

**Method**

**Participants**

Participants’ inclusion criteria were: (1) male gender, (2) age between 12-16 years old, (3) normal or corrected to normal vision, (4) no diagnosis of ADHD or epilepsy. Only male participants were selected in order to avoid gender as a possible confound factor in the pain judgement task (Han et al., 2008), and to overcome gender differences in video gaming habits (Krahé & Möller, 2010; Rideout, 2015). In line with the institutional ethical guidelines, participants below 16 years old provided consent together with their parents, and participants aged 16 years old provided their consent individually.

**Individual Characteristics**

***Video Gaming Habits*** were measured with two questions about the average number of hours spent on video gaming (1) on a weekday and (2) on a weekend day, answered on a scale from 1 = “0 hours” to 8 = “11 hours or more” (Rideout et al., 2010; den Hamer et al., 2017).

***Trait empathy*** was measured with the Interpersonal Reactivity Index (IRI; Davis, 1983) in a Dutch translation by De Corte et al., (2007). The full scale was applied (α = .74), including 28 items. Sample item: ‘I often have tender, concerned feelings for people less fortunate than me’. Participants answered on a scale ranging from 1 (*does not describe me at all*) to 5 (*describes me very well*). A mean of the IRI scale was calculated (Table S1).

***Trait physical aggressiveness*** was measured by the Buss-Perry Aggression Questionnaire (BPAQ; Buss & Perry, 1992) in Dutch translation by Konijn et al. (2007). The subscale consisted of 9 items (α = .84), for example: ‘I get into fights a little more than the average person’. Scale ranged from 1 (*does not describe me at all*) to 5 (*describes me very well*). A mean of the BPAQ subscale was calculated (Table S1).

***Trait sensation-seeking*** was measured with five items (α = .89): two by Stephenson et al. (2003), and three items added in a Dutch translation by den Hamer et al. (2017), e.g., ‘I wish my life was more exciting’. Scale ranged from 1 (*does not describe me at all*) to 5 (*describes me very well*). A mean of the scale was calculated (Table S1).

**Video Gameplay Experience Check**

Participants indicated how they experienced the game based on six questions, each followed by a 7-point rating scale (1 = not at all / strongly disagree, and 7 = extremely / strongly agree). They were asked about their perceived level of (1) violence in the game, (2) frustration caused by the game, (3) excitement during the game, (4) engagement in the game, (5) interest in the game, and (6) game challenge (Engelhardt et al., 2015).

**Analysis Plan**

Following the analytical approach of Miedzobrodzka et al. (2022b), in case of a significant Pain x Time x VVGE interaction, the main analysis was followed by a secondary analysis with the same within-subject factors and also including the VVGE group (low VVGE vs. high VVGE) as a between-subjects factor (instead of habitual VVGE as a covariate)^[[1]](#footnote-1)^.

**Results**

**Individual Characteristics**

Six participants were left-handed (10.7%). Most of participants played video games for 1 (30.4%) or 2 hours (28.6%) in a weekday and 3-4 hours (42.9%) in the weekend.

**Correlations**

Correlation analysis between the main individual characteristics indicated that neither VVGE, nor non-VVGE were related to any of the individual difference measures. However, we observed that exposure to antisocial media content (C-ME) was positively related with trait aggressiveness (*r* = .50; *p* < .01) and with trait sensation seeking (*r* = .52; *p* < .01). Finally, a positive relationship between trait aggressiveness and sensation seeking was found (*r* = .56; *p* < .01). Moreover, behavioral pain ratings for painful pictures were positively related to trait empathy (both pre-game: *r* = .32; *p* = .022, and post-game: *r* = .33; *p* = .016). Detailed results for individual differences and behavioural pain ratings are presented in Table S2. Details of correlations with video gameplay experience check are presented in Table S3.

***ERP Results***

Based on the preregistered protocol, we excluded participants who had less than 24 trials in one or more of the four ERP categories. This resulted in exclusion of three participants from the pre-game condition, and three participants from the post-game condition. Moreover, post-game EEG data of one participant were not recorded due to a software failure. This resulted in *N* = 53 in the pre-game condition, *N* = 52 in the post-game condition, and *N* = 50 who had complete EEG data from both conditions and who were used for the ERP analyses.

**Exploratory Analyses**

Equation for the repeated measures moderation, with the difference between ERP amplitudes for painful pictures pre-game vs. post-game as a dependent variable (YDi), and with C-ME as a moderator (W):

𝑌_𝑖1_=𝑏_01_+𝑏_1_𝑊_𝑖_+𝜖_𝑖1_

𝑌_𝑖2_=𝑏_02_+𝑏_2_𝑊_𝑖_+𝜖_𝑖2_

𝑌_𝑖2_−𝑌_𝑖1_=𝑏_02_−𝑏_01_+(𝑏_12_−𝑏_11_)𝑊_𝑖_+𝜖_𝑖2_−𝜖_𝑖1_

𝑌_𝐷𝑖_=𝑏_0_+𝑏_1_𝑊_𝑖_+𝜖_𝑖_

For details, please see Montoya, (2019).

**Table S1**

*Means and Standard Deviations of the Individual Characteristics (N = 56)*

| Variable | *M* (*SD*) |
| --- | --- |
| 1. VVGE | 9.46 (7.80) |
| 2. non-VVGE | 1.88 (4.59) |
| 3. C-ME anti | 2.25 (0.74) |
| 4. IRI Empathy | 2.78 (0.37) |
| 5. BPAQ PA | 2.11 (0.67) |
| 6. SS | 2.33 (1.03) |

*Note.* Standard deviations are presented in parentheses. VVGE = Violent Video Game Exposure; non-VVGE = Non-violent Video Game Exposure; C-ME anti = Antisocial subscale of Content-based Media Exposure scale; IRI Empathy = Trait Empathy; BPAQ PA = Trait Physical Aggressiveness; SS = Trait Sensation Seeking.

**Table S2**

*Correlations between Measures of Individual Differences and Behavioral Pain Ratings (N = 56)*

| Variable | 1 | | 2 | | 3 | | 4 | | 5 | | 6 | | 7 | | 8 | | 9 | |
| --- | --- | --- | --- | --- | --- | --- | --- | --- | --- | --- | --- | --- | --- | --- | --- | --- | --- | --- |
| 1. VVGE |  | — | –.06 |  | .25 |  | .03 |  | .10 |  | .07 |  | .13 |  | .16 |  | .10 |  |
| 2. non-VVGE |  |  | — |  | –.02 |  | –.07 |  | –.07 |  | –.11 |  | –.10 |  | –.16 |  | –.15 |  |
| 3. C-ME anti |  |  |  |  | — |  | .21 |  | .50 | *** | .52 | *** | .21 |  | .13 |  | .10 |  |
| 4. IRI Empathy |  |  |  |  |  |  | — |  | –.10 |  | .02 |  | .03 |  | .32 | * | .33 | * |
| 5. BPAQ PA |  |  |  |  |  |  |  |  | — |  | .56 | *** | .10 |  | –.17 |  | –.13 |  |
| 6. SS |  |  |  |  |  |  |  |  |  |  | — |  | .13 |  | –.05 |  | –.03 |  |
| 7. Age |  |  |  |  |  |  |  |  |  |  |  |  | — |  | –.02 |  | –.03 |  |
| 8. Pain Pre |  |  |  |  |  |  |  |  |  |  |  |  |  |  | — |  | .90 | *** |
| 9. Pain Post |  |  |  |  |  |  |  |  |  |  |  |  |  |  |  |  | — |  |

*Note.* * *p* < .05; ** *p* < .01; *** *p* < .001; VVGE = Violent Video Game Exposure; non-VVGE = Non-violent Video Game Exposure;

C-ME anti = Antisocial subscale of Content-based Media Exposure scale; IRI Empathy = Trait Empathy; BPAQ = Trait Physical Aggressiveness; SS = Trait Sensation Seeking; Pain Pre = Behavioral Pain Rating Pre-Game Condition; Pain Post = Behavioral Pain Rating Post-Game Condition.

**Table S3**

*Correlations between Measures of Gameplay Experience Check and the habitual exposure to video games measures (N = 56)*

| Variable | 1 | | 2 | | 3 | | 4 | | 5 | | 6 | | 7 | | 8 | |
| --- | --- | --- | --- | --- | --- | --- | --- | --- | --- | --- | --- | --- | --- | --- | --- | --- |
| 1. VVGE |  | — | –.05 |  | .20 |  | –.16 |  | .01 |  | .02 |  | .08 |  | –.05 |  |
| 2. non-VVGE |  |  | — |  | –.20 |  | –.13 |  | –.09 |  | –.05 |  | .07 |  | –.05 |  |
| 3. violence |  |  |  |  | — |  | –.08 |  | .12 |  | .45 | ** | .16 |  | .41 | ** |
| 4. frustration |  |  |  |  |  |  | — |  | .06 |  | .06 |  | .02 |  | –.18 |  |
| 5. excitement |  |  |  |  |  |  |  |  | — |  | .04 |  | .35 | ** | –.19 |  |
| 6. engagement |  |  |  |  |  |  |  |  |  |  | — |  | .07 |  | .26 |  |
| 7. interest |  |  |  |  |  |  |  |  |  |  |  |  | — |  | –.08 |  |
| 8. challenge |  |  |  |  |  |  |  |  |  |  |  |  |  |  | — |  |

*Note.* * *p* < .05; ** *p* < .01; *** *p* < .001; VVGE = Violent Video Game Exposure; non-VVGE = Non-violent Video Game Exposure.

**Exploratory results**

**Table S4**

*Main and interaction effects for the P3 component with VVGE and age as covariates*

| Effect | *df* | *F* | *p* | *η_p_^2^* |
| --- | --- | --- | --- | --- |
| 1. Time | 1,46 | 1.14 | .292 | .024 |
| 2. Pain | 1,46 | 2.06 | .159 | .043 |
| 3. Time x Pain | 1,46 | < 0.01 | .998 | < .001 |
| 4. Time x Age | 1,46 | 1.05 | .311 | .022 |
| 5. Time x VVGE | 1,46 | 0.13 | .178 | .003 |
| 6. Pain x Age | 1,46 | 1.71 | .197 | .036 |
| 7. Pain x VVGE | 1,46 | 3.21 | .080 | .065 |
| 8. Time x Pain x Age | 1,46 | < 0.01 | .991 | < .001 |
| 9. Time x Pain x VVGE | 1,46 | 0.21 | .649 | .005 |
| 10. Pain x Age x VVGE | 1,46 | 3.37 | 0.73 | .068 |
| 11. Time x Age x VVGE | 1,46 | 0.12 | .732 | .003 |

*Note.* VVGE = Violent Video Game Exposure.

**Table S5**

*Main and interaction effects for the LPP component with VVGE and age as covariates*

| Effect | *df* | *F* | *p* | *η_p_^2^* |
| --- | --- | --- | --- | --- |
| 1. Time | 1,46 | 0.71 | .402 | .015 |
| 2. Pain | 1,46 | 2.32 | .134 | .048 |
| 3. Time x Pain | 1,46 | 0.74 | .393 | .016 |
| 4. Time x Age | 1,46 | 0.49 | .486 | .011 |
| 5. Time x VVGE | 1,46 | 0.20 | .889 | < .001 |
| 6. Pain x Age | 1,46 | 0.79 | .379 | .017 |
| 7. Pain x VVGE | 1,46 | 3.02 | .089 | .062 |
| 8. Time x Pain x Age | 1,46 | 0.65 | .424 | .014 |
| 9. Time x Pain x VVGE | 1,46 | 0.02 | .882 | < .001 |
| 10. Pain x Age x VVGE | 1,46 | 2.85 | 0.098 | .058 |
| 11. Time x Age x VVGE | 1,46 | 0.01 | .925 | < .001 |

*Note.* VVGE = Violent Video Game Exposure.

**Table S6**

*Main and interaction effects for the P3 component with C-ME and age as covariates*

| Effect | *df* | *F* | *p* | *η_p_^2^* |
| --- | --- | --- | --- | --- |
| 1. Time | 1,47 | 1.82 | .184 | .037 |
| 2. Pain | 1,47 | 0.02 | .888 | < .001 |
| 3. Time x Pain | 1,47 | 0.20 | .659 | .004 |
| 4. Time x Age | 1,47 | 0.78 | .381 | .016 |
| 5. Time x C-ME | 1,47 | 2.70 | .107 | .054 |
| 6. Pain x Age | 1,47 | 0.03 | .854 | .001 |
| 7. Pain x C-ME | 1,47 | 0.07 | .793 | .001 |
| 8. Time x Pain x Age | 1,47 | 1.01 | .321 | .021 |
| 9. Time x Pain x C-ME | 1,47 | 5.43 | .024 | .104 |

*Note.* C-ME = Content-based Media Exposure, indicating level of exposure to antisocial media content.

**Table S7**

*Main and interaction effects for the LPP component with C-ME as a covariate*

| Effect | *df* | *F* | *p* | *η_p_^2^* |
| --- | --- | --- | --- | --- |
| 1. Time | 1,48 | 3.19 | .080 | .062 |
| 2. Pain | 1,48 | 14.47 | < .001 | .232 |
| 3. C-ME | 1,48 | 2.55 | .117 | .051 |
| 5. Time x Pain | 1,48 | 0.88 | .354 | .018 |
| 6. Time x C-ME | 1,48 | 1.58 | .215 | .032 |
| 7. Pain x C-ME | 1,48 | 0.18 | .677 | .004 |
| 8. Time x Pain x C-ME | 1,48 | 2.11 | .153 | .042 |

*Note.* C-ME = Content-based Media Exposure, indicating level of exposure to antisocial media content.

**Table S8**

*Main and interaction effects for the LPP component with C-ME and age as covariates*

| Effect | *df* | *F* | *p* | *η_p_^2^* |
| --- | --- | --- | --- | --- |
| 1. Time | 1,47 | 1.44 | .236 | .030 |
| 2. Pain | 1,47 | 0.10 | .759 | .002 |
| 3. Time x Pain | 1,47 | 1.23 | .272 | .026 |
| 4. Time x Age | 1,47 | 0.58 | .450 | .012 |
| 5. Time x C-ME | 1,47 | 1.17 | .284 | .024 |
| 6. Pain x Age | 1,47 | 0.50 | .485 | .010 |
| 7. Pain x C-ME | 1,47 | 0.30 | .589 | .006 |
| 8. Time x Pain x Age | 1,47 | 1.98 | .166 | .040 |
| 9. Time x Pain x C-ME | 1,47 | 2.92 | .094 | .058 |

*Note.* C-ME = Content-based Media Exposure, indicating level of exposure to antisocial media content.

**Figure S1**

*Distribution of habitual Violent Video Game Exposure (VVGE) across the whole sample (N = 56)*

**

**Figure S2**

*Standard electrodes’ placement*


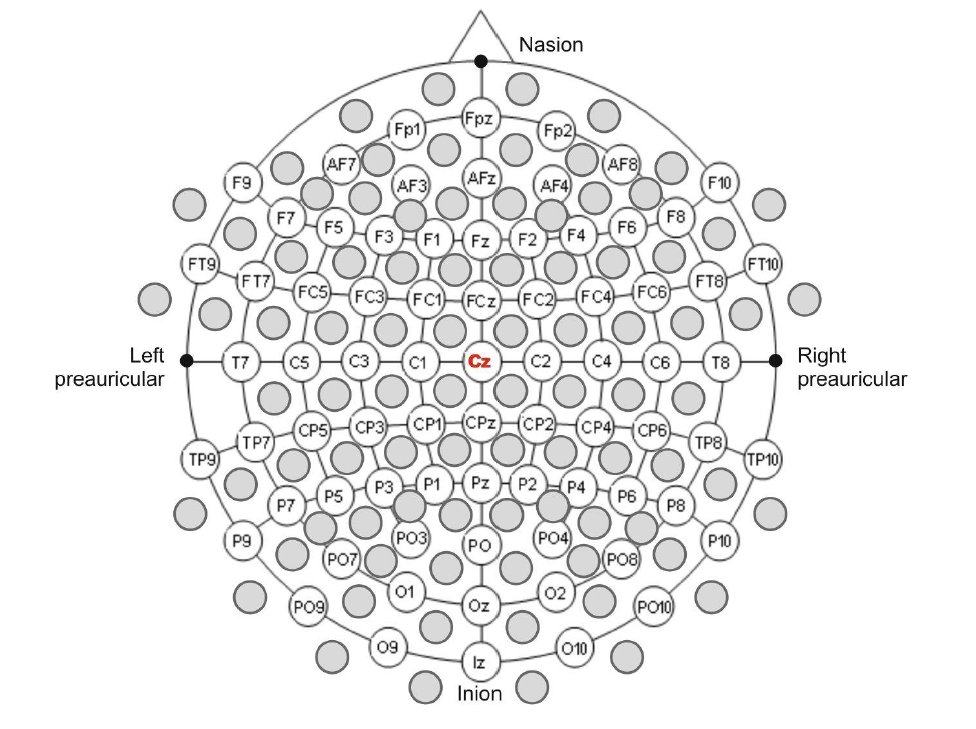


*Note.* Marked 6 electrodes (red circles) used for analyses of P3 and LPP: CP1, CPz, CP2, P1, Pz, P2. Two electrodes were placed at mastoids and were used as a reference.

**References**

Buss, A. H., & Perry, M. P., (1992). The Aggression Questionnaire. *Journal of Personality and Social Psychology*, *63*, 452–459. <https://doi.org/10.1037/0022-3514.63.3.452>

Davis, M. H. (1983). Measuring individual differences in empathy: Evidence for a multidimensional approach. *Journal of Personality and Social Psychology, 44*, 113–126. <https://doi.org/10.1037/0022-3514.44.1.113>

De Corte, K., Buysse, A., Verhofstadt, L.L., Roeyers, H., Ponnet, K., & Davis, M.H. (2007). Measuring Empathic Tendencies: Reliability and Validity of the Dutch Version of the Interpersonal Reactivity Index. *Psychologica Belgica*, *47*, 235–260. <http://doi.org/10.5334/pb-47-4-235>

den Hamer, A., Konijn, E., & Bushman, B. (2017). Measuring Exposure to Media with Antisocial and Prosocial Content: An Extended Version of the Content-based Media Exposure Scale (C-ME2). *Communication Methods and Measures*, *11*, 289–299. <https://doi.org/10.1080/19312458.2017.1375089>

Engelhardt, C., Mazurek, M., Hilgard, J., Rouder, J., & Bartholow, B. (2015). Effects of violent-video-game exposure on aggressive behavior, aggressive-thought accessibility, and aggressive affect among adults with and without autism spectrum disorder. *Psychological Science*, *26*, 1187–1200. <https://doi.org/10.1177/0956797615583038>

Han, S., Fan, Y., & Mao, L. (2008). Gender difference in empathy for pain: An electrophysiological investigation. *Brain Research, 1196*, 85–93. <https://doi.org/10.1016/j.brainres.2007.12.062>

Konijn, E. A., Nije Bijvank, M., & Bushman, B. J. (2007). I wish I were a warrior: The role of wishful identification in the effects of violent video games on aggression in adolescent boys. *Developmental Psychology, 43*, 1038–1044. <https://doi.org/10.1037/0012-1649.43.4.1038>

Krahé, B., & Möller, I. (2010). Longitudinal effects of media violence on aggression and empathy among German adolescents. *Journal of Applied Developmental Psychology*, *31*, 401–409. <https://doi.org/10.1016/j.appdev.2010.07.003>

Montoya A. K. (2019). Moderation analysis in two-instance repeated measures designs: Probing methods and multiple moderator models. *Behavior Research Methods, 51*(1), 61–82. <https://doi.org/10.3758/s13428-018-1088-6>

Rideout, V. (2015). The Common Sense Census: Media Use by Tweens and Teens. Common Sense Media, San Francisco. Retrieved from: <https://www.commonsensemedia.org/sites/default/files/uploads/research/census_researchreport.pdf>

Rideout, V. J., Foehr, U. G., & Roberts, D. F. (2010). Generation M2: Media in the lives of 8-to 18-year-olds. Menlo Park, CA: Kaiser Family Foundation. <http://kff.org/other/report/generation-m2-media-in-the-lives-of-8-to-18-year-olds/>

Stephenson, M. T., Hoyle, R. H., Palmgreen, P., & Slater, M. D. (2003). Brief measures of sensation seeking for screening and large-scale surveys. *Drug and Alcohol Dependence*, *72*(3), 279–286. <https://doi.org/10.1016/j.drugalcdep.2003.08.003>

1. The condition of the follow-up analysis (a significant Pain x Time x VVGE interaction) was not preregistered. [↑](#footnote-ref-1)
